# Discovery of four plasmatic biomarkers potentially predicting cardiovascular outcome in peripheral artery disease

**DOI:** 10.1038/s41598-022-23260-3

**Published:** 2022-11-01

**Authors:** B. M. M. Kremers, J. N. Posma, S. Heitmeier, J. Glunz, H. ten Cate, A. Pallares Robles, J. H. C. Daemen, A. J. ten Cate-Hoek, B. M. E. Mees, H. M. H. Spronk

**Affiliations:** 1 Laboratory for Clinical Thrombosis and Hemostasis, Department of Biochemistry, Cardiovascular Research Institute Maastricht, Maastricht University, Universiteitssingel 50, Room 4.330, 6229 ER Maastricht, The Netherlands; 2 Bayer AG, Wuppertal, Germany; 3 Thrombosis Expertise Center and Department of Internal Medicine, Maastricht University Medical Center, Maastricht, The Netherlands; 4 Center for Thrombosis and Hemostasis, University Medical Center of the Johannes Gutenberg-University, Mainz, Germany; 5 Department of Vascular Surgery, Maastricht University Medical Center, Maastricht, The Netherlands

**Keywords:** Proteomics, Predictive markers, Atherosclerosis, Peripheral vascular disease, Thrombosis

## Abstract

Peripheral artery disease (PAD) patients have an increased cardiovascular risk despite pharmacological treatment strategies. Biomarker research improving risk stratification only focused on known atherothrombotic pathways, but unexplored pathways might play more important roles. To explore the association between a broad cardiovascular biomarker set and cardiovascular risk in PAD. 120 PAD outpatients were enrolled in this observational cohort study. Patients were followed for one year in which the composite endpoint (myocardial infarction, coronary revascularization, stroke, acute limb ischemia and mortality) was assessed. Patient data and blood samples were collected upon inclusion, and citrated platelet-poor plasma was used to analyze 184 biomarkers in Olink Cardiovascular panel II and III using a proximity extension assay. Fifteen patients reached the composite endpoint. These patients had more prior strokes and higher serum creatinine levels. Multivariate analysis revealed increased plasma levels of protease-activated receptor 1 (PAR1), galectin-9 (Gal-9), tumor necrosis factor receptor superfamily member 11A (TNFRSF11A) and interleukin 6 (IL-6) to be most predictive for cardiovascular events and mortality. Positive regulation of acute inflammatory responses and leukocyte chemotaxis were identified as involved biological processes. This study identified IL-6, PAR1, Gal-9, TNFRSF11A as potent predictors for cardiovascular events and mortality in PAD, and potential drug development targets.

## Introduction

Peripheral artery disease (PAD) involves atherosclerotic plaque formation in peripheral vascular beds leading to progressive blood flow restriction in large and medium-sized arteries. PAD patients are at increased risk of atherothrombotic events such as myocardial infarction, ischemic stroke or cardiovascular death, with an incidence of 5% to 14% each year^1^. This high incidence of cardiovascular events within PAD populations is partly caused by concomitantly affected vascular beds, such as the coronary arteries, in more than 60% of all PAD patients^2,3^. The rate of complications illustrates the need to better identify patients at highest risk that would benefit from more intensive cardiovascular risk management. Although several biomarkers have been identified as predictors of cardiovascular events and mortality in previous studies, as summarized in our recent systematic review^4^, surprisingly none have been implemented yet in clinical management. One reason is the perception that current biomarkers including high-sensitivity c-reactive protein (hs-CRP), neutrophil–lymphocyte ratio (NLR), fibrinogen, d-dimer, N-terminal pro brain natriuretic peptide (NT-proBNP) and high-sensitivity cardiac troponin T (hs-cTnT) still lack power to tailor individual patient management. In other vascular diseases, like atrial fibrillation, the ABC-score comprising two biomarkers NT-proBNP and hs-cTnT, can be used to estimate stroke risk^5^. Pursuing a similar strategy in PAD patients should start with searching candidate biomarkers with the potential to identify patients with an increased cardiovascular risk. We therefore decided to explore a broad set of cardiovascular biomarkers from different biological processes that were not known to be associated with risk stratification in PAD.

## Results

Baseline characteristics for the whole cohort are shown in Table 1. All 120 patients completed the one-year follow-up, although 4 patients were unable to give blood and could therefore not be included in the biomarker analysis. The cohort comprised 70 (58.3%) male patients with an average age of 67.7 (± 9.6) years. Most patients had symptoms of intermittent claudication (88 (73.3%)) while the remaining 32 (26.7%) had chronic limb threatening ischemia. Many patients had a prior PAD revascularization 84 (70%) while 39 (32.5%) had a prior myocardial infarction and another 14 (11.7%) suffered from a prior stroke. The average BMI was 26.5 (± 4.5) kg/m^2^ and 53 (44.2%) patients were current smokers upon inclusion. DM2 was diagnosed in 31 (25.8%) patients and the median plasma creatinine level was 90 (74–105 μmol/L).

**Table 1:** Baseline characteristics for the whole cohort and distribution between patients with and without cardiovascular events during follow-up. Significance was reached when *p* < 0.05 (*).

	Total cohort	Event group	No event group	*P* value
	Mean ± SD/Median (IQR) /n (%)	Mean ± SD/Median (IQR) /n (%)	Mean ± SD /Median (IQR)/n (%)	
Age (years)	67.7 ± 9.6	72.1 ± 7.7	67.1 ± 9.7	0.056
Male gender	70 (58.3)	8 (53.3)	62 (59)	0.440
Prior myocardial infarction	39 (32.5)	8 (53.3)	31 (29.5)	0.064
Prior ischemic stroke	14 (11.7)	5 (33.3)	9 (8.6)	0.016*
Prior PAD revascularization	84 (70)	12 (80)	72 (68.9)	0.281
Current smoking	53 (44.2)	8 (53.3)	45 (42.9)	0.312
Body Mass Index (kg/m^2^)	26.5 ± 4.5	27.1 ± 5.2	26.4 ± 4.5	0.557
Diabetes Mellitus type 2	31 (25.8)	5 (33.3)	26 (24.8)	0.335
Creatinine (µmol/l)	90 (74–105)	97 (81–154)	86 (73–104)	0.036*
Antiplatelet drugs	120 (100)	15 (100)	105 (100)	1.000
Lipid-lowering drugs	115 (95.8)	13 (86.7)	102 (97.1)	0.058
Antihypertensive drugs	90 (75)	11 (73.3)	79 (75.2)	0.908
**Rutherford classification**				0.090
Rutherford 1	9 (7.5)	1 (6.7)	8 (7.6)	
Rutherford 2	34 (28.3)	2 (13.3)	32 (30.5)	
Rutherford 3	45 (37.5)	4 (26.7)	41 (39)	
Rutherford 4	32 (26.7)	8 (53.3)	24 (22.9)	

Significant values are in bold.

Within the whole cohort 15 patients suffered from an event during follow-up with recordings of 9 myocardial infarctions, 3 elective coronary interventions, 2 ischemic strokes and 1 death. Age and gender did not differ between patients with and without events, however more prior strokes were observed in patients with events (5 (33.3%) vs. 9 (8.6%), *p* = 0.016). Although not significant, more prior myocardial infarctions were observed in patient with events (8 (53.3% vs. 31 (29.5%), *p* 0.064). Kidney function was significantly worse in patients with events (plasma creatinine 97 (81–154) μmol/L vs. 86 (73–104) μmol/L, *p* = 0.036) while other cardiovascular risk factors were similar between the groups (Table 1).

**Figure 1:**
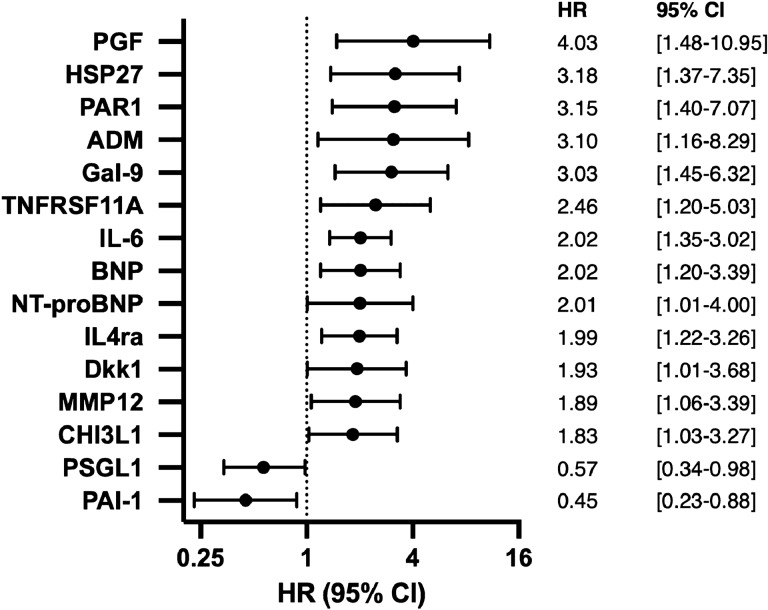
Biomarkers with a significant positive or negative predictive value for cardiovascular events and mortality, identified by multivariate cox regression analysis.

All biomarkers were added to the multivariate cox regression analysis with correction for age, gender, prior myocardial infarction or stroke and creatinine levels, revealing 13 proteins to be positively predictive for cardiovascular events and mortality. Placenta growth factor (PGF) appeared to have the highest hazard ratio (HR [95% Confidence interval (CI)]) (HR 4.03 [1.48–10.95]) followed by heat shock protein 27 (HSP; HR 3.18 [1.37–7.35]), protease-activated receptor 1 (PAR1; HR 3.15 [1.40–7.07]), adrenomedullin (ADM; 3.10 [1.16–8.29]), galectin-9 (Gal-9; HR 3.03 [1.45–6.32]), tumor necrosis factor superfamily member 11A (TNFRSF11A; HR 2.46 [1.20–5.03]), interleukin-6 (IL-6; HR 2.02 [1.35–3.02]), brain natriuretic peptide (BNP; HR 2.02 [1.20–339]), N-terminal pro brain natriuretic peptide (NT-proBNP; HR 2.01 [1.01–4.00]), interleukin-4 receptor subunit alpha (IL4ra; HR 1.99 [1.22–3.26]), Dickkopf-related protein 1 (Dkk1; HR 1.93 [1.01–3.68]), matrix metalloproteinase-12 (MMP12; HR 1.89 [1.06–3.39]) and chitinase-3-like protein 1 (CHI3L1; HR 1.83 [1.03–3.27]). Another 2 proteins were negatively predictive for cardiovascular events and mortality, being p-selectin glycoprotein ligand 1 (PSGL1; HR 0.57 [0.34–0.98]) and plasminogen activator inhibitor 1 (PAI-1; HR 0.45 [0.23–0.88]) (Fig. 1).

**Figure 2:**
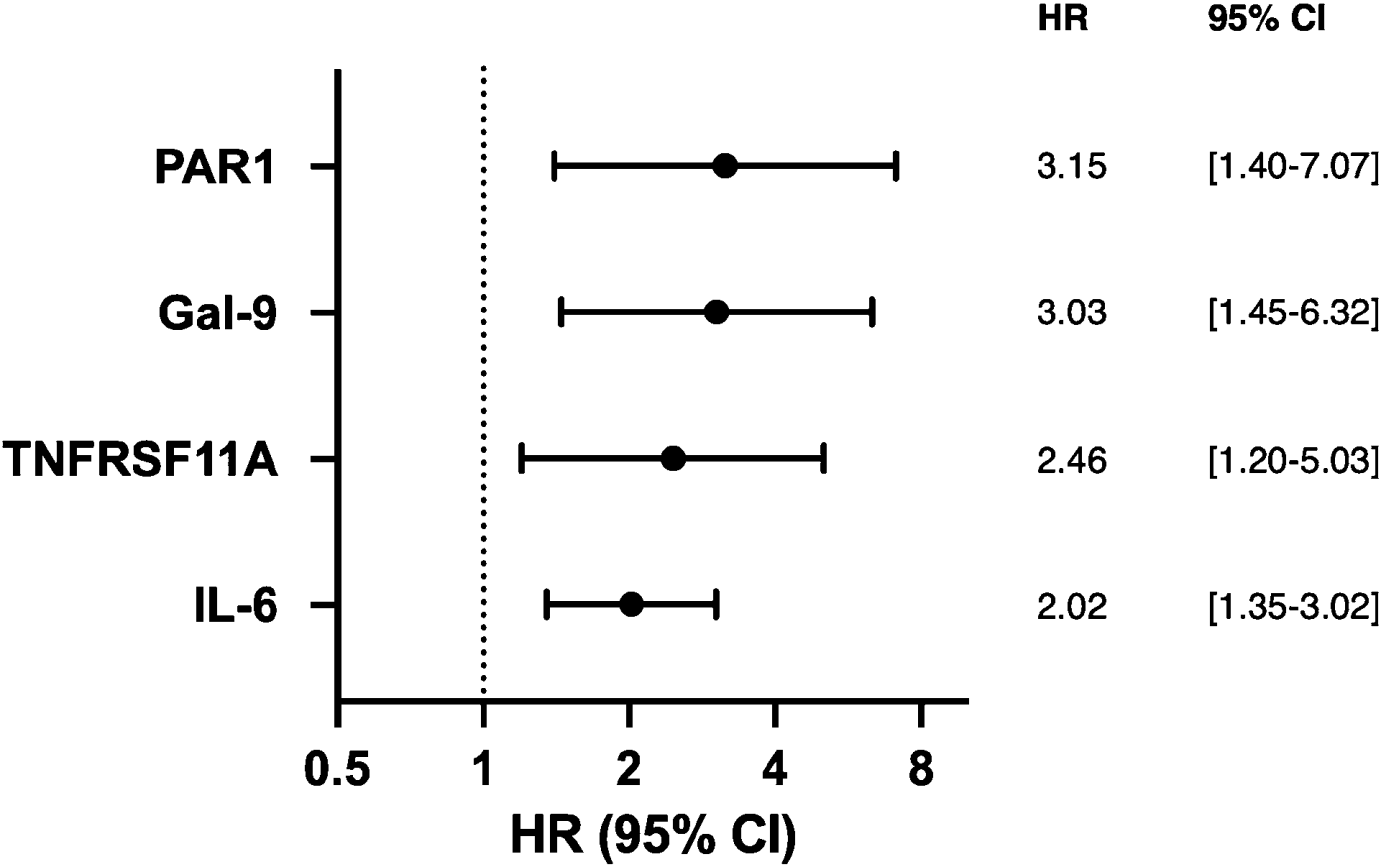
Biomarkers with significantly increased plasma levels in patients with events, identified by multivariate cox regression analysis with LASSO.

To identify the most predictive biomarkers across all proteins, LASSO regression analysis was performed (C-index 0.84) that revealed protease-activated receptor 1 (PAR1), galectin-9 (Gal-9), tumor necrosis factor receptor superfamily member 11A (TNFRSF11A) and interleukin 6 (IL-6) as most predictive biomarkers for cardiovascular events and mortality (Fig. 2). PAR1 showed the highest predictive value with a hazard ratio of 3.15 [1.40–7.07] followed by Gal-9 (HR 3.03 [1.45–6.32]), TNFRSF11A (HR 2.46 [1.20–5.03] and IL-6 (2.02 [1.35–3.02]).

## Discussion

Patients with PAD are at increased risk of (recurrent) cardiovascular events and death despite current treatment strategies. Biomarkers may potentially help to identify patients with different risk profiles. The main finding of our study is that four proteins (IL-6, PAR1, TNFRSF11A and Gal-9) are linked to cardiovascular outcomes in PAD patients and therefore are novel candidate biomarkers for risk assessment in PAD.

The pro-inflammatory cytokine IL-6 induces the synthesis and release of several acute phase proteins such as C-reactive protein (CRP) and fibrinogen^6^. IL-6 has a variety of functions, making it a key player in the inflammatory response, also in different stages of atherosclerosis. In early stages, IL-6 coordinates influx of inflammatory cells into atherosclerotic lesions. IL-6 increases expression of intercellular adhesion molecule 1 (ICAM-1) and thereby enables leukocytes to be recruited and transmigrated into the vessel wall^7^. IL-6 has also been shown to increase the surface expression of tissue factor on cultured monocytes, thereby initiating the coagulation cascade^8^. In later stages of atherosclerosis, growth and progression of atherosclerotic lesions are promoted by IL-6 through induction of platelet-derived growth factor (PDGF) which causes growth of vascular smooth muscle cells^9^. In the final stages of atherosclerosis, including atherothrombosis, IL-6 induces aggregation and activation of platelets and thereby accelerates thrombus formation. Aggregation of platelets is stimulated through the production of fibrinogen^10^ while activation is enhanced through increased expression of P-selectin^11^. Combining these functions, IL-6 appears to play an important pro-atherogenic role throughout all stages of atherosclerosis, including thrombus formation and arterial occlusion. This important role is also seen in clinical studies, where IL-6 is a significant predictor of PAD progression independently of traditional cardiovascular risk factors. Moreover, IL-6 is predictive of changes in ankle-brachial index, thereby illustrating a change in the degree of atherosclerosis^12^. Surprisingly, IL-6 has not been investigated in regard to the occurrence of cardiovascular events in PAD populations specifically. Studies in other populations, such as patients with prior stroke and polyvascular disease, show an increased risk of recurrent stroke in patients with elevated IL-6 levels^13^.

PAR1 is a membrane-bound protein mostly found on endothelial cells, platelets and vascular smooth muscle cells. As PAR1 is a transmembrane receptor, measured plasma concentrations of soluble PAR1 do not represent functional receptor. However, PAR1 is known to be internalized by multivesicular bodies (MVB). Cargo from MVBs can be degraded by lysosomes or secreted as exosomes. This suggests that PAR1 concentrations in plasma might reflect exosome PAR1 concentrations as a marker of cellular receptor processing. Furthermore, PAR1 plasma levels can also reflect cell death^14^. Thrombin is the main activator of PAR1^15^ and given the increased thrombin production in subjects with atherosclerosis, this may also result in further upregulation of PAR1 receptor expression^16^. PAR1, like IL-6, plays an important role throughout various stages of atherosclerosis. In early stages, matrix metalloproteinase-9 (MMP-9)-mediated PAR1 activation induces endothelial dysfunction leading to a loss of vascular integrity^17^. PAR1 on endothelial cells can also be activated by activated protein C (APC) upregulating monocyte chemotactic protein 1 (MCP-1). MCP1 promotes not only pro-inflammatory effects, but also anti-inflammatory effects, indicating anti-atherogenic effects of PAR1 activation^18,19^. In later stages, pro-atherothrombotic effects become more visible as activation of PAR1 on platelets leads to platelet activation through thromboxane A2 production and aggregation through P-selectin upregulation^20,21^. Overall, activation of PAR1 induces mostly pro-atherosclerotic effects and thereby increases the cardiovascular risk, making it a potent predictive plasma biomarker. PAR1 has not been investigated thoroughly as a potential biomarker, but is well-known for its selective inhibition by vorapaxar. The TRA2P-TIMI 50 trial demonstrated a significant benefit of vorapaxar in reducing cardiovascular death and ischemic events in patients with a history of acute myocardial infarction, ischemic stroke or peripheral artery disease^22^.

TNFRSF11A is commonly known as receptor activator of nuclear factor kappa-B (RANK) and is part of the RANK/RANKL/OPG signaling pathway which regulates osteoclast differentiation and activation^23^. Activation of NF-kB is usually mediated by RANK ligand, however overexpression of RANK itself is sufficient to activate this pathway^24^. Via NF-kB signaling, endothelial cells become activated and express various chemokines (e.g. MCP1) and adhesion molecules (e.g. ICAM-1) responsible for chemotaxis and transmigration of leukocytes into atherosclerotic plaques^25^. Progression and evolvement of plaques is further stimulated through NF-kB by accumulation and proliferation of VSMCs^26^. In late stages of atherosclerosis, NF-kB plays an important role in regulating activation and aggregation of platelets however underlying mechanisms remain to be elucidated^27^. TNFRSF11A has not been investigated as biomarker in association with progression of atherosclerosis in PAD or the occurrence of cardiovascular events.

Gal-9 is an important immune regulator which is abundantly present in several chronic inflammatory diseases such as inflammatory bowel disease^28^ and systemic lupus erythematosus^29^. Expressed by many different cell types such as endothelial cells, macrophages, and T-lymphocytes^30^, Gal-9 is thought to be anti-inflammatory via TIM-3 signaling. Important effects of this signaling include apoptosis of pro-inflammatory Th1 and Th17 cells^30,31^ and stimulation of regulatory T cell activity^32^, both to dampen atherosclerotic progression. Serum levels of Gal-9 were found to be decreased in patients with coronary artery disease, specifically those with acute coronary syndrome. However, other studies reported higher serum Gal-9 levels in patients with DM2 and chronic kidney disease, two morbidities that were abundantly present in our cohort^33^. Gal-9 seems to be anti-inflammatory and thus atherosclerosis-dampening, however certain factors such as kidney function and the presence of DM2 may alter levels of Gal-9^33^. Therefore, Gal-9 should be used with caution and the presence of co-morbidities should be considered. As with TNFRSF11A, Gal-9 has not been investigated as biomarker in association with progression of atherosclerosis in PAD or the occurrence of cardiovascular events.

PSGL1 and PAI-1 were negatively predictive for cardiovascular events and mortality, indicating that increased levels of these biomarkers are associated with fewer events. PSGL1 plays an important role as inflammatory marker in atherogenesis with involvement in leukocyte recruitment and activation. Studies investigating the role of PSGL1 in atherosclerosis mostly found accelerating effects of PSGL1 on the atherosclerotic process. The absence of PSGL1 in PSGL^-/-^ transgenic mice reduced atherosclerotic plaque surface area, inflammatory cell infiltration and hyperplasia^34^. Clinical studies investigating PSGL1 are limited, but *Ozaki *et al*.* reported that expression levels of PSGL1 on monocytes were high in acute coronary syndrome patients^35^. The increased levels of PSGL1 in association with fewer events in our study may be explained by the function of PSGL1 as it activates intracellular protein kinases^36^. Hereby atherosclerosis may be accelerated, but not necessarily lead to the formation of unstable plaques. A similar mechanism may explain the increased levels of PAI-1, which appeared to be protective of events in our cohort. While a recent systematic review found an association between increased PAI-1 levels and the occurrence of cardiovascular events, lower PAI-1 levels were associated with increased restenosis^37^. Although both PSGL1 and PAI-1 appeared protective in our study, other studies have found positive associations between these biomarkers and the occurrence of events, which in part can be explained by variations in pro-inflammatory status and other cardiovascular risk factors^38,39^. It remains to be elucidated what the exact roles of PSGL1 and PAI-1 are in the process of atherosclerosis.

This study has several limitations. First of all, all biomarkers were measured in Olink panels using the Proximity Extension Assay (PEA) technology followed by PCR. This semiquantitative technology provides relative concentrations of a biomarker in plasma rather than absolute concentrations. Therefore, these results must be validated in a quantitative assay to measure quantitative concentrations. Furthermore, our sample size was limited to 120 patients, yielding higher confidence intervals in several biomarker hazard ratio calculations. Lastly, patients were only followed for 12 months in total, limiting the total event rate.

In conclusion, risk stratification models in PAD patients are necessary to predict future cardiovascular events and death. This study identified IL-6, PAR1, TNFRSF11A and Gal-9 as promising biomarkers to aid in risk stratification. These proteins are involved in prominent atherosclerotic biological processes including activation of endothelial cells, positive regulation of acute inflammatory responses, leukocyte chemotaxis and platelet activation. This semi-quantitative biomarker discovery is a first step to improve risk stratification in PAD. The next step would be to perform quantitative assays to confirm the association with cardiovascular outcome, preferably in a separate PAD population.

## Methods

### Study design

Outpatients of the department of Vascular Surgery of the Maastricht University Medical Center (MUMC +) were screened for PAD between 2018 and 2020. Eligibility for study participation was based on the ankle-brachial index, which had to be 0.9 or below. Within the selection of patients with an abnormal ankle-brachial index, we selected patients with Rutherford 1–2–3/Fontaine IIa-IIb (intermittent claudication) or Rutherford 4/Fontaine III (chronic limb threatening ischemia). Patients with Rutherford 5–6/Fontaine IV were not eligible due to increased inflammatory parameters rising from ulcer formation or tissue loss. Active malignancy, chronic inflammatory disease, coagulation disorders or anticoagulant therapy, pregnancy and age below eighteen were other exclusion criteria. All eligible patients willing to participate were included after written informed consent was obtained. Upon inclusion, patient characteristics were collected and blood was drawn from the patient. All patients were followed for one year in which the primary outcome was assessed. The Medical Ethics Committee of the MUMC + approved the study (NL63235.068.17) and the study was registered in the Netherlands Trial Register (NTR7250; https://www.trialregister.nl/trial/7045). All experiments were performed in accordance with the relevant guidelines and regulations.

### Blood collection and sample storage

Venous blood was drawn upon inclusion by antecubital venipuncture with 21-gauge needles and 3.2% (w/v) citrated Vacutainer glass tubes. The blood collection tubes were immediately processed using the standard platelet-poor plasma centrifugation (4000×*g* for 5 min and 11,000×*g* for 10 min). After centrifugation, the plasma aliquots were frozen and stored at − 80 °C.

### Data collection and outcome

Patient characteristics were recorded at baseline including gender and age of each patient as well as a history of myocardial infarction, ischemic stroke and PAD revascularization. Each patient provided an updated medication list from which the use of lipid-lowering drugs, antihypertensive drugs and antiplatelet drugs were collected. Information on the presence of traditional risk factors smoking, renal insufficiency, diabetes mellitus type 2 (DM2) and body mass index (BMI) was also obtained. Kidney function was evaluated by measuring plasma creatinine levels. The outcome of the study comprised a composite endpoint of myocardial infarction, stroke, acute limb ischemia, elective percutaneous coronary intervention or coronary artery bypass grafting and all-cause mortality during one year of follow-up. Outcome verification took place by a combination of telephone calls to the patient and analysis of hospital records.

### Biomarker analysis

Citrated platelet-poor plasma was used to measure protein concentrations using the ProSeek Cardiovascular II and III panels (Olink Biosciences, Uppsala, Sweden). These panels are based on proximity extension assay (PEA) technology allowing simultaneous measurements of 92 protein biomarkers per panel. In total, 184 different proteins were measured in each patient. Pairs of oligonucleotide-labeled antibodies bind pair-wise to target proteins present in 1 μL of plasma, leading to the formation of a new polymerase chain reaction (PCR) target sequence formed by a proximity-dependent DNA polymerization event. The resulting sequence is subsequently detected and quantified by standard real-time PCR. Measurements are specified as Normalized Protein Expression (NPX), generated from the PCR quantification cycles. NPX data are then used to establish protein signatures where high NPX values equal high proteins concentrations and low NPX values equal low proteins concentrations.

### Statistical analysis

Statistical analysis was performed on the whole cohort and a comparison was made between patients who reached the composite endpoint (event group) and patients who did not (no event group). Differences in baseline characteristics for continuous variables were presented as mean with standard deviation or median with interquartile range, as appropriate. Dichotomous and categorical variables were defined as frequencies with percentages and compared using the Fisher’s Exact test or chi-square testing, while continuous variables were compared using the parametric two-samples *t *test or the non-parametric Mann–Whitney U test. Protein expression levels following non-normal distributions were transformed into normal distributions using logarithmic transformation. Missing values were imputed to prevent a loss of statistical precision and to reduce the likelihood of selection bias, using random forest imputation, implemented in the R package ‘missForest’^40^. The relation of the standardized biomarker levels (mean = 0 and SD = 1) with the cardiovascular outcome was assessed using individual Cox Hazard proportional regression models adjusted for age, gender, prior myocardial infarction, prior stroke and plasma creatinine levels. Then, to identify a subset of best predictive biomarkers for cardiovascular events and mortality during follow-up, LASSO regression analysis was performed with a tenfold cross validation to increase generalizability of the models (glmnet package^41^). The selected biomarkers were shown as hazard ratios and 95% confidence intervals from previous individual Cox regression models. Due to the explorative nature of this study, a nominal *p* value < 0.05 was used to reach statistical significance. All analyses were performed using R (R Core Team (2013) version 3.5.3. R: A language and environment for statistical computing. R Foundation for Statistical Computing, Vienna, Austria).

### Ethics declarations

The Medical Ethics Committee of the MUMC + approved the study (NL63235.068.17).

## Data Availability

Data will be available upon reasonable request by B. Kremers.
